# Retinal Diseases and Parkinson Disease: A Population-Based Study

**DOI:** 10.3389/fnins.2021.679092

**Published:** 2021-08-30

**Authors:** Po-Chih Chen, Chen-Chih Chung, Yun-Yung Cheng, Wan-Ting Chen, Chien-Tai Hong, Lung Chan, Li-Nien Chien

**Affiliations:** ^1^Department of Neurology, Shuang-Ho Hospital, Taipei Medical University, New Taipei City, Taiwan; ^2^Department of Neurology, School of Medicine, College of Medicine, Taipei Medical University, Taipei City, Taiwan; ^3^Graduate Institute of Biomedical Informatics, Taipei Medical University, Taipei City, Taiwan; ^4^School of Health Care Administration, College of Management, Taipei Medical University, Taipei City, Taiwan; ^5^Health and Clinical Research Data Center, Office of Data, Taipei Medical University, Taipei City, Taiwan

**Keywords:** retina, Parkinson’s disease, population-based, cohort study, dopamine

## Abstract

**Introduction:**

Patients with Parkinson disease (PD) tend to have ophthalmic symptoms. Retinal diseases are associated with central nervous system diseases, especially neurodegenerative diseases. Here, we investigated the association of retinal diseases with PD, especially the temporal relationship before and after PD diagnosis.

**Methods:**

Data were obtained from the National Health Insurance Research Database of Taiwan. In total, 21,845 patients with newly diagnosed PD were matched with four controls each on the basis of propensity score. This study was bidirectional. A case–control study evaluated the adjusted odds ratio (aOR) of retinal disease before PD diagnosis by using conditional logistic regression. Furthermore, a cohort study evaluated the adjusted subdistribution hazard ratio (aSHR) for new-onset retinal and optic nerve diseases after PD diagnosis by using competing risk analysis. The association between PD with optic nerve diseases and glaucoma (another common ophthalmic diseases with the consequence of retinal dysfunction) were also analyzed as reference.

**Results:**

In the case–control study, PD was found to be significantly comorbid with recent and remote retinal disease [recent: ≤ 5 years, aOR: 1.12, 95% confidence interval (CI): 1.03–1.23; remote: > 5 years, aOR: 1.18, 95% CI: 1.04–1.34]. No similar association was identified between optic nerve disease or glaucoma with PD. In the cohort study, patients with PD were found to have a low risk of retinal disease in short-term (≤ 5 years, aSHR: 0.81, 95% CI: 0.71–0.93) and long-term (> 5 years, aSHR: 0.82, 95% CI: 0.72–0.93) follow-up.

**Conclusion:**

The study findings demonstrated that patients with prediagnostic PD were at greater risk of retinal disease than non-PD participants, but the risk reversed afterward. Thus, retinal disease may be a premotor manifestation of PD, and there may be some possible effect of dopamine supplements on retina.

## Introduction

Parkinson disease (PD) is the second most common neurodegenerative disease worldwide, with an estimated incidence of 15–328 per 100,000 individuals and prevalence of 15–12,500 per 100,000 individuals. The disease prevalence has been increasing over time, affecting roughly 2.5 million patients in 1990 and 6.1 million patients (5.0–7.3) in 2016 (GBD 2016 Parkinson’s Disease Collaborators, 2018). Among PD risk factors, age is the most important and un-modified. With progress in the domains of public health and medicine as well as increased life expectancy (Christensen et al., 2009) among the general population, the number of patients with PD will likely continue to increase in the future.

Motor symptoms in PD are characteristic and include resting tremor, bradykinesia, postural instability, and freezing phenomenon. Non-motor symptoms at various stages of PD include ophthalmologic symptoms and disorders (Borm et al., 2020), mood disorders and affective apathy, anhedonia and depression, cognitive dysfunction, complex behavioral disorders, and hallucinations (Poewe, 2008).

Despite advances in technology and modern medicine, the actual mechanism of PD remains obscure. The disease is characterized by dopaminergic neuron depletion and abnormal intracellular α-synuclein aggregation in Lewy bodies. Both environmental (Klingelhoefer and Reichmann, 2015) and genetic factors (Koros et al., 2017), such as neuroinflammation, mitochondrial dysfunction (Rocha et al., 2018), and characteristics of the brain–gut axis, appear to be associated with PD pathogenesis, although none of them can explain the disease phenomenon completely.

Dopaminergic neuron depletion, associated with the shortage of dopamine up to approximately 70%, results in the manifestation of clinical motor symptoms of PD (Engelender and Isacson, 2017). Dopamine is a neurotransmitter with key roles in not only the central nervous system (CNS) but also the gastrointestinal system (Glavin and Szabo, 1990), immune response mediation (Matt and Gaskill, 2020), light adaptation (Flood et al., 2018), and eye growth (Stone et al., 1989).

Visual disturbances are prominent at all PD stages (Satue et al., 2017), with up to 82% of patients reporting dry eyes, blepharitis, double vision, or visual hallucination (Borm et al., 2020). The shortage and depletion of dopamine have been associated with the retinal degeneration and denervation of the visual cortex or adjacent regions in animal studies and clinical studies involving patients with PD (Phillipson et al., 1987; Weil et al., 2016). In an animal study, dopaminergic treatment preserved visual function (Pardue and Allen, 2018). However, levodopa and dopamine agonists used in the treatment of PD are associated with ocular and visual adverse effects (Armstrong, 2017; Daggumilli et al., 2019).

Given the high prevalence of ophthalmologic symptoms in patients with PD (Satue et al., 2017) and the possible role of dopamine in retinal functions, we investigated the association of retinal diseases with PD. To test the role of dopamine as a premotor PD biomarker and avoid the possible bias introduced by dopaminergic medications, we conducted case–control and cohort studies using patient data from the National Health Insurance Research Database (NHIRD). The bidirectional approach helped assess the risk of newly diagnosed retinal disease before and after PD diagnosis. To minimize the potential of identifying retinal diseases secondary to glaucoma or optic nerve disease, a prevalent disease in the elderly population, we analyzed the risk of glaucoma and optic nerve diseases in parallel for the purpose of comparison (Weinreb et al., 2014).

## Materials and Methods

### Data Source

Patient data were obtained from the NHIRD, which is maintained by the National Health Insurance (NHI) Administration (NHIA) of Taiwan. The NHIRD is a nationwide claims-based database of those insured under the NHI program, which is a compulsory insurance program that has been providing coverage for most of the health care services in Taiwan and almost 30,000 prescription medications since 1995. In this study, we used data collected between 2000 and 2017, and data collected after 2000 were used because electronic claims data were incomplete during the initial phases of NHI implementation. The NHIRD includes information on disease diagnoses [coded according to the *International Classification of Diseases, Ninth Revision, Clinical Modification* (ICD-9-CM) before 2016 and according to ICD-10 thereafter], treatment procedures, service dates, prescribed medications (classified according to the Anatomical Therapeutic Chemical Classification System for Medications), reimbursement amounts, patient demographic information, and patient- and provider-encrypted identifiers. To verify the accuracy of diagnoses and treatment rationales, the NHIA routinely samples a portion of the NHI claims and penalizes hospitals and clinics if they determine unnecessary medical treatment has been provided.

### Study Population

Patients with newly diagnosed PD were defined as those who had at least two diagnostic claims (ICD-9-CM: 332.0) and prescription claims for dopaminergic agents between 2004 and 2013. It had been validated that the diagnostic accuracy of this inclusion criteria was 94.8% (Lee et al., 2013). The index date of PD was defined as the date of first PD diagnosis, hereafter referred to as the index PD. Patients aged < 45 years or who had a history of stroke or prior treatment with an antipsychotic drug before the index PD were excluded to avoid the possibility of misclassification of secondary parkinsonism. In addition, patients with a history of thyroid disease were excluded, as thyroid dysfunction (ICD-9-CM: 240–246) may be directly associated with ophthalmic diseases. The same exclusion criteria were used for control participants.

### Propensity Score Matching

Matching aims to reduce potential selection bias in observational studies. Propensity score (PS) matching (PSM) is frequently used to control for confounding factors that inevitably occur in studies investigating the effect of exposures on an outcome. In PSM, study and control groups sharing similar propensity scores are matched. The weighted value reveals the risk of a participant for the outcome of interest according to underlying characteristics that predispose them to that outcome irrespective of the exposure of interest. In this study, the PS was measured on the basis of hypertension (HTN, ICD-9-CM: 401–405), diabetes mellitus (DM, ICD-9-CM: 250), hyperlipidemia (ICD-9-CM: 272), chronic heart failure (CHF, ICD-9-CM: 428), coronary artery disease (CAD, ICD-9-CM: 410–414), chronic lung disease (ICD-9-CM: 415–417, 490–496, and 500–508), renal disease (ICD-9-CM: 580–589), and inflammatory diseases (ICD-9-CM: 710, 714). The selection of these factors was based on their association with retinal and optic nerve diseases. Control participants without PD were assigned an index date of pseudo-PD diagnosis corresponding to the index PD of their matched patients. Each patient with PD was matched with four control participants without PD based on age, sex, pseudo diagnostic year, and the PS using a caliper with a width of 0.1; consequently, the two cohorts had similar baseline characteristics but differed in PD diagnosis.

### Main Outcome

**FIGURE 1:**
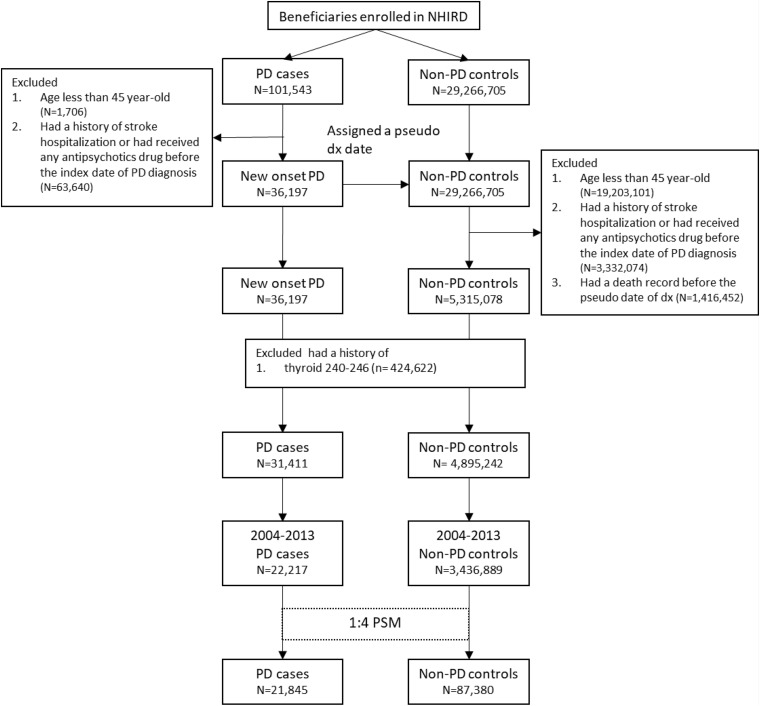
Flowchart of patient selection.

Both patients with PD and control participants were tracked or followed up for their risk of retinal and optic nerve diseases according to the study design. Patients with retinal diseases (ICD-9-CM: 361–363 except 363.4–363.7) were defined as those who first had at least two diagnostic claims corresponding with the fundus examination. In the cohort study, retinal disease risk was measured after the index PD or pseudo-PD diagnosis. Hereditary retinal disease (ICD-9-CM: 362.7) were excluded. Optic nerve disease was defined as the presence of two disease diagnostic claim (ICD-9-CM: 377) and traumatic optic nerve disease (ICD-9-CM:377.3) was excluded. Glaucoma was defined as the presence of disease diagnosis (ICD-9-CM:365) and medication treatment. The detailed disease diagnostic codes are presented in Supplementary Table 1. The selection process is presented in Figure 1.

### Statistical Analysis

Baseline characteristics were analyzed using the standardized mean difference (SMD). An SMD of > 0.1 indicated non-negligible differences between the groups. The case–control study evaluated the adjusted odds ratio (aOR) of retinal disease before PD diagnosis by using conditional logistic regression, and the cohort study evaluated the adjusted subdistribution hazard ratio (aSHR) of new-onset retinal and optic nerve diseases after PD diagnosis by using competing risk analysis. Competing risk model analyses were applied to estimate the absolute relative retinal disease risks because the participants had a high mortality risk. The follow-up period for each patient ranged from the index PD or pseudo-PD diagnosis to the date of retinal and optic nerve disease diagnosis, death, or the end of the observation period (December 31, 2017). All analyses were performed using SAS/STAT version 9.4 (SAS Institute Inc., Cary, NC, United States) and STATA 14 (Stata Corp., LP, College Station, TX, United States). A *p-*value of < 0.05 was considered significant.

## Results

Figure 1 is the flow chart detailing the selection of patients with PD. Overall, 21,845 patients with newly diagnosed PD and 87,380 non-PD PS-matched control participants for comparison were included. No differences were present in age or sex between the groups (Table 1). Comorbidities, including DM, hyperlipidemia, CHF, CAD, chronic lung disease, renal disease, and inflammatory diseases, were well-matched between the PD and non-PD control groups.

**TABLE 1:** Baseline characteristics of participants with PD versus non-PD before and after PSM.

	Before matching					After matching				
	Non-PD		PD		SMD	Non-PD		PD		SMD
	n	(%)	n	(%)		n	(%)	n	(%)	
Sample size	3,436,889		22,217			87,380		21,845		
Male	1,917,573	(55.8)	13,139	(59.1)	0.068	51,712	(59.2)	12,928	(59.2)	< 0.001
**Age, years**										
Mean (SD)	57.67	(10.44)	72.05	(9.89)	1.414	71.89	(9.82)	71.89	(9.82)	< 0.001
45–64	2,639,180	(76.8)	4,857	(21.9)	1.315	19,386	(22.2)	4,847	(22.2)	< 0.001
65+	797,709	(23.2)	17,360	(78.1)	1.315	67,994	(77.8)	16,998	(77.8)	< 0.001
**Year of diagnosis**										
2004–2005	651,760	(19.0)	4,306	(19.4)	0.011	16,936	(19.4)	4,234	(19.4)	< 0.001
2006–2007	698,620	(20.3)	4,558	(20.5)	0.005	17,884	(20.5)	4,471	(20.5)	< 0.001
2008–2009	698,440	(20.3)	4,474	(20.1)	0.005	17,568	(20.1)	4,392	(20.1)	< 0.001
2010–2011	693,020	(20.2)	4,470	(20.1)	0.001	17,620	(20.2)	4,405	(20.2)	< 0.001
2012–2013	695,049	(20.2)	4,409	(19.8)	0.009	17,372	(19.9)	4,343	(19.9)	< 0.001
**Comorbidities**										
HTN	732,817	(21.3)	11,127	(50.1)	0.629	46,891	(53.7)	10,796	(49.4)	0.085
DM	332,270	(9.7)	4,687	(21.1)	0.321	17,404	(19.9)	4,552	(20.8)	0.023
Hyperlipidemia	353,140	(10.3)	4,036	(18.2)	0.227	13,730	(15.7)	3,950	(18.1)	0.063
CHF	32,781	(1.0)	898	(4.0)	0.199	3,427	(3.9)	819	(3.7)	0.009
CAD	179,610	(5.2)	3,902	(17.6)	0.396	14,913	(17.1)	3,713	(17.0)	0.002
Chronic lung disease	145,698	(4.2)	2,682	(12.1)	0.289	10,953	(12.5)	2,520	(11.5)	0.031
Renal disease	51,413	(1.5)	1,107	(5.0)	0.198	3,893	(4.5)	1,038	(4.8)	0.014
Inflammatory disease	27,063	(0.8)	338	(1.5)	0.069	984	(1.1)	324	(1.5)	0.031
Statin prescription	59,571	(4.6)	2,108	(9.5)	0.190	7,577	(8.7)	2,057	(9.4)	0.026
Average clinic visits/year	10.32	(11.83)	23.65	(17.49)	0.893	21.86	(15.93)	22.79	(15.94)	0.058

**Standardized mean difference (SMD) indicates the variable difference in means or proportions divided by standard error; imbalance defined as absolute value > 0.1. PD, Parkinson disease; PSM, propensity score matching; HTN, hypertension; DM, diabetes mellitus; CHF, congestive heart failure; CAD, coronary heart disease.**

The overall risk of retinal disease was significantly increased in the PD group (aOR: 1.14, 95% CI: 1.06–1.23, *p* < 0.001) compared with the non-PD group before PD diagnosis. The significantly increased risk was consistent following separation of the premotor stage into recent (≤ 5 years; aOR: 1.12, 95% CI: 1.03–1.23) and remote (> 5 years; aOR: 1.18, 95% CI: 1.04–1.34) periods. The increased risk was not evident in the diagnosis of optic nerve disease or glaucoma, other common ophthalmic diseases with the consequence of retinal disease, between the groups before PD diagnosis (Table 2 and Supplementary Tables 2, 3).

**TABLE 2:** The aOR of retinal disease, optic nerve disease and glaucoma among the study participants in the case–control study.

	PD	Non-PD	aOR (95% CI)	*p*-value
Participants	21,845	87,380		
**Retinal disease, n (%)**				
*Overall*	972 (4.4)	3,428 (3.9)	1.14 (1.06–1.23)	<0.001
Recent (≤5 years)	654 (3.0)	2,344 (2.7)	1.12 (1.03–1.23)	0.011
Remote (>5 years)	318 (1.5)	1,084 (1.2)	1.18 (1.04–1.34)	0.011
**Optic Nerve disease, n (%)**				
*Overall*	86 (0.4)	275 (0.3)	1.25 (0.98–1.60)	0.069
Recent (≤5 years)	60 (0.3)	211 (0.2)	1.14 (0.85–1.51)	0.379
Remote (>5 years)	26 (0.1)	64 (0.1)	1.63 (1.03–2.58)	0.036
**Glaucoma, n (%)**				
*Overall*	1,363 (6.2)	5,157 (5.9)	1.06 (1.00–1.13)	0.057
Recent (≤5 years)	695 (3.2)	2,645 (3.0)	1.05 (0.97–1.15)	0.234
Remote (>5 years)	668 (3.1)	2,512 (2.9)	1.07 (0.98–1.16)	0.146

**aOR, adjusted odds ratio; PD, Parkinson disease; CI, confidence interval.**

Regarding the follow-up cohort, we excluded participants with a diagnosis of any ophthalmologic disorder before PD diagnosis to focus on newly diagnosed retinal diseases after PD diagnosis. The results demonstrated a significant reduction in the hazard ratio of newly diagnosed retinal disease (aSHR: 0.77, 95% CI: 0.70–0.85, *p* < 0.001), and the reduced risk remained significant in short-term (≤ 5 years; aSHR: 0.81, 95% CI: 0.71–0.93) and long-term (> 5 years; aSHR: 0.82, 95% CI: 0.72–0.93) follow-up. Regarding optic nerve disease, the overall hazard ratio was not different between PD and non-PD (aSHR: 0.91, 95% CI: 0.67–1.23, *p* = 0.540). For the glaucoma, the overall hazard ratio was significantly reduced (aSHR: 0.87, 95% CI: 0.78–0.97, *p* = 0.010) but was identical between PD and non-PD groups at short-term (≤ 5 years) follow-up and significantly lower only in the PD group at long-term (> 5 year) follow-up (Table 3 and Supplementary Tables 4, 5).

**TABLE 3:** The aSHR of retinal disease, optic nerve disease and glaucoma among participants in the cohort study.

	PD	Non-PD	aOR (95% CI)	*p*-value
**Retinal disease, n/N (%)**				
*Overall*	446/11,184 (4.3)	2,465/45,986 (5.7)	0.77 (0.70–0.85)	<0.001
Short-term (≤5 years)	216/11,184 (1.9)	1,137/45,986 (2.5)	0.81 (0.71–0.93)	0.003
Long-term (>5 years)	230/7,928 (2.9)	1,328/35,308 (3.8)	0.82 (0.72–0.93)	0.003
**Optic nerve disease, n/N (%)**				
*Overall*	50/11,184 (0.4)	226/45,986 (0.5)	0.91 (0.67–1.23)	0.540
Short-term (≤5 years)	23/11,184 (0.2)	105/45,986 (0.2)	0.94 (0.60–1.47)	0.770
Long-term (>5 years)	27/8,086 (0.3)	1,231/36,193 (0.3)	1.05 (0.70–1.60)	0.799
**Glaucoma, n/N (%)**				
*Overall*	340/11,184 (3.0)	1,611/45,986 (3.5)	0.87 (0.78–0.97)	0.010
Short-term (≤5 years)	216/11,184 (1.9)	880/45,986 (1.9)	1.04 (0.90–1.20)	0.567
Long-term (>5 years)	124/7,924 (1.6)	731/35,500 (2.1)	0.75 (0.62–0.89)	0.001

**aSHR, adjusted subdistribution hazard ratio; PD, Parkinson disease; CI, confidence interval.**

## Discussion

The present study demonstrated that patients with PD are at higher risk of retinal disease at the premotor PD stage than non-PD controls, although the hazard ratio reversed markedly in the follow-up period. This contrasting association was not observed between PD and optic nerve disease or glaucoma, other common age-related ophthalmic diseases. This discrepancy in the temporal relationship between the two diseases may hint that retinal disease is a premotor manifestation of PD, and the possible effect of dopamine supplements on retina.

Anosmia is a well-recognized non-motor symptom of the premotor PD stage (Iannilli et al., 2017), and the degeneration of the olfactory bulb is found to occur before the loss of dopaminergic neurons in the midbrain. Similar to the olfactory bulb, the optic nerve and retina are considered to be extension of the CNS (London et al., 2013). Dopamine is endogenously found in and essential for the functioning of the retina, although disorders of these structures are markedly underestimated in patients with PD. Pathological α-synuclein aggregation and deposits have been noted in retinal cells in postmortem patients with PD (Veys et al., 2019). Thinning of the retinal nerve fiber layers, a measure of the integrity of the retinal ganglion cell axon, has been found in PD, and macular thickness has also been reported to be reduced (Altintaş et al., 2008). The association between PD with retinal disease suggest possible explanation that dopaminergic deficiency is harmful for the retina of key to maintain structural integrity (Witkovsky, 2004). Applying dopamine was found to be effective in slowing retinal degeneration in some preclinical studies and clinical trials (Zhou et al., 2017) and the possible benefit of dopamine supplement on restoring visual and neuronal function were the stimulation of the secretion of pigment epithelium derived factor, and anti-angiogenesis (Review by Pardue and Allen, 2018). The present study used a bidirectional approach to successfully distinguish the reverse association between the two diseases; our approach is superior to previous cross-sectional, case–control studies because it could discern the temporal relationship and avoid the bias from dopaminergic supplements.

Glaucoma is widely recognized as an age-related disease and a leading cause of retinal and optic nerve disease. The present study did not find an increased risk of glaucoma for patients with PD in the prediagnostic stage, and this excluded the possible bias of increased risk of retinal and optic nerve disease secondary to uncontrolled glaucoma. Regarding the follow-up period for patients with PD, we found a significant reduction of the hazard ratio of newly diagnosed glaucoma. Previous studies have shown the effects of a dopamine receptor agonist in decreasing intraorbital pressure over several hours (Pescosolido et al., 2013), but there is scant evidence of a more prolonged effect. We speculate that supplementation with dopaminergic agents may lower the incidence of glaucoma in patients with PD in the long-term, although further studies must investigate the actual mechanism through which this could be achieved.

To the best of our knowledge, this is the first study to elucidate the association of retinal disease with PD using a bidirectional approach and the first to identify a discrepancy in risk based on temporal association. The main strengths of our study are attributable to the characteristics of the cohort. The NHIRD contains comprehensive data on a nationwide population (> 99% of Taiwan’s population), and these data have been collected for over two decades. Typically, the diagnosis of PD and retinal and optic nerve disease is made by specialists. Because we used the NHIRD, we were able to take advantage of data encompassing long prediagnostic and follow-up periods before and after PD diagnosis. Moreover, the data were free from the false recall concern, which is a common concern in most case–control studies, and the percentage of loss to follow-up in the cohort was low. Despite these advantages, the present study has certain limitations. First, the NHIRD does not have information regarding family history of PD, environmental factors, or occupational factors, all of which may affect the incidence of retinal degeneration or PD. To minimize potential bias, we excluded patients diagnosed with PD before 45 years of age, but this exclusion criterion could not eliminate all genetic-related PD. Second, the severity of retinal and optic nerve disease was not documented in the NHIRD, which limits further analysis of the diseases with PD risk. Third, although the finding of significant risk reduction of retinal disease after the diagnosis of PD, as the nature of epidemiological study, it was not able to clearly delineate the causal relationship between dopamine supplement with the risk reduction, which may need more studies to confirm. Lastly, in the NHIRD, there was no information abouts the motor symptoms and motor subtypes of PD, which limited the possibility of further subgroup analysis.

In conclusion, the study demonstrated that patients with PD are at a greater risk of retinal disease at the prediagnostic stage, even more than 5 years in advance of diagnosis, than are non-PD individuals. By contrast, the risk of retinal disease after PD diagnosis decreased significantly compared with the control group, implying the possible effect from dopaminergic supplements. Further large-scale prospective studies investigating retinal and optic nerve disease as a premotor predictive biomarker for PD are warranted.

## Data Availability Statement

The raw data supporting the conclusions of this article will be made available by the authors, without undue reservation.

## Ethics Statement

This study was approved by the Joint Institutional Review Board of Taipei Medical University (approval N202101060). Confidentiality was ensured by adhering to data regulations of the Health and Welfare Data Science Center (HWDC), Ministry of Health and Welfare, Executive Yuan, Taiwan. To protect patient privacy, individual identifiers are encrypted before the HWDC releases patient-level data to investigators, which they do only for research purposes. Therefore, the requirement for informed consent from study participants was exempted by the Joint Institutional Review Board. All study methods were in accordance with guidelines approved by the Joint Institutional Review Board and aforementioned governmental regulations.

## Author Contributions

L-NC, LC, and C-TH: conceptualization, formal analysis, writing—review and editing. P-CC, C-CC, Y-YC, W-TC, LC, and L-NC: data curation. P-CC, C-CC, Y-YC, W-TC, and C-TH: methodology. LC and L-NC: supervision and validation. C-CC: visualization. P-CC, C-CC, and Y-YC: writing—original draft. All authors contributed to the article and approved the submitted version.

## Conflict of Interest

The authors declare that the research was conducted in the absence of any commercial or financial relationships that could be construed as a potential conflict of interest.

## Publisher’s Note

All claims expressed in this article are solely those of the authors and do not necessarily represent those of their affiliated organizations, or those of the publisher, the editors and the reviewers. Any product that may be evaluated in this article, or claim that may be made by its manufacturer, is not guaranteed or endorsed by the publisher.
