# Supplementary material for: Retinal Diseases and Parkinson Disease: A Population-Based Study
Source: Front Neurosci. 2021 Aug 30;15:679092. doi: 10.3389/fnins.2021.679092 (PMC8435857; doi:10.3389/fnins.2021.679092)
Supplement: Supplementary file 1 [file Table_1.DOCX]

Supplementary Information

Supplementary Table 1. The detailed diagnostic code of retinal disease, optic nerve disease and glaucoma

|  | ICD9 | ICD10 |
| --- | --- | --- |
| Retinal disease | 361-363  except 362.7 and 363.4-363.7 | E11311, E11319, E11321, E11329, E11331, E11339, E11341, E11349, E11351, E11359, G453, H30001, H30002, H30003, H30009, H30011, H30012, H30013, H30019, H30021, H30022, H30023, H30029, H30031, H30032, H30033, H30039, H30041, H30042, H30043, H30049, H30101, H30102, H30103, H30109, H30111, H30112, H30113, H30119, H30121, H30122, H30123, H30129, H30131, H30132, H30133, H30139, H30141, H30142, H30143, H30149, H3020, H3021, H3022, H3023, H30811, H30812, H30813, H30819, H30891, H30892, H30893, H30899, H3090, H3091, H3092, H3093, H31001, H31002, H31003, H31009, H31011, H31012, H31013, H31019, H31021, H31022, H31023, H31029, H31091, H31092, H31093, H31099, H32, H33001, H33002, H33003, H33009, H33011, H33012, H33013, H33019, H33021, H33022, H33023, H33029, H33031, H33032, H33033, H33039, H33041, H33042, H33043, H33049, H33051, H33052, H33053, H33059, H33101, H33102, H33103, H33109, H33111, H33112, H33113, H33119, H33191, H33192, H33193, H33199, H3320, H3321, H3322, H3323, H33301, H33302, H33303, H33309, H33311, H33312, H33313, H33319, H33321, H33322, H33323, H33329, H33331, H33332, H33333, H33339, H3340, H3341, H3342, H3343, H338, H3400, H3401, H3402, H3403, H3410, H3411, H3412, H3413, H34211, H34212, H34213, H34219, H34231, H34232, H34233, H34239, H34811, H34812, H34813, H34819, H34821, H34822, H34823, H34829, H34831, H34832, H34833, H34839, H349, H3500, H35011, H35012, H35013, H35019, H35021, H35022, H35023, H35029, H35031, H35032, H35033, H35039, H35041, H35042, H35043, H35049, H35051, H35052, H35053, H35059, H35061, H35062, H35063, H35069, H35071, H35072, H35073, H35079, H3509, H35101, H35102, H35103, H35109, H35111, H35112, H35113, H35119, H35121, H35122, H35123, H35129, H35131, H35132, H35133, H35139, H35141, H35142, H35143, H35149, H35151, H35152, H35153, H35159, H35161, H35162, H35163, H35169, H35171, H35172, H35173, H35179, H3520, H3521, H3522, H3523, H3530, H3531, H3532, H3533, H35341, H35342, H35343, H35349, H35351, H35352, H35353, H35359, H35361, H35362, H35363, H35369, H35371, H35372, H35373, H35379, H35381, H35382, H35383, H35389, H3540, H35411, H35412, H35413, H35419, H35421, H35422, H35423, H35429, H35431, H35432, H35433, H35439, H35441, H35442, H35443, H35449, H35451, H35452, H35453, H35459, H35461, H35462, H35463, H35469, H3560, H3561, H3562, H3563, H3570, H35711, H35712, H35713, H35719, H35721, H35722, H35723, H35729, H35731, H35732, H35733, H35739, H3581, H3582, H3589, H359, H36 |
| optic nerve disease | 377  Except 377.3 | H2000, H20011, H20012, H20013, H20019, H20021, H20022, H20023, H20029, H20031, H20032, H20033, H20039, H20041, H20042, H20043, H20049, H20051, H20052, H20053, H20059, H2010, H2011, H2012, H2013, H2020, H2021, H2022, H2023, H20811, H20812, H20813, H20819, H20821, H20822, H20823, H20829, H209, H2100, H2101, H2102, H2103, H211X1, H211X2, H211X3, H211X9, H21211, H21212, H21213, H21219, H21221, H21222, H21223, H21229, H21231, H21232, H21233, H21239, H21241, H21242, H21243, H21249, H21251, H21252, H21253, H21259, H21261, H21262, H21263, H21269, H21271, H21272, H21273, H21279, H2129, H21301, H21302, H21303, H21309, H21311, H21312, H21313, H21319, H21321, H21322, H21323, H21329, H21341, H21342, H21343, H21349, H21351, H21352, H21353, H21359, H2140, H2141, H2142, H2143, H21501, H21502, H21503, H21509, H21511, H21512, H21513, H21519, H21521, H21522, H21523, H21529, H21531, H21532, H21533, H21539, H21541, H21542, H21543, H21549, H21551, H21552, H21553, H21559, H21561, H21562, H21563, H21569, H2181, H2182, H2189, H219, H22, H4040X0 |
| glaucoma | 365 | H40001, H40002, H40003, H40009, H40011, H40012, H40013, H40019, H40021, H40022, H40023, H40029, H40031, H40032, H40033, H40039, H40041, H40042, H40043, H40049, H40051, H40052, H40053, H40059, H40061, H40062, H40063, H40069, H4010X0, H4010X1, H4010X2, H4010X3, H4010X4, H4011X0, H4011X1, H4011X2, H4011X3, H4011X4, H401210, H401211, H401212, H401213, H401214, H401220, H401221, H401222, H401223, H401224, H401230, H401231, H401232, H401233, H401234, H401290, H401291, H401292, H401293, H401294, H401310, H401311, H401312, H401313, H401314, H401320, H401321, H401322, H401323, H401324, H401330, H401331, H401332, H401333, H401334, H401390, H401391, H401392, H401393, H401394, H401410, H401411, H401412, H401413, H401414, H401420, H401421, H401422, H401423, H401424, H401430, H401431, H401432, H401433, H401434, H401490, H401491, H401492, H401493, H401494, H40151, H40152, H40153, H40159, H4020X0, H4020X1, H4020X2, H4020X3, H4020X4, H40211, H40212, H40213, H40219, H402210, H402211, H402212, H402213, H402214, H402220, H402221, H402222, H402223, H402224, H402230, H402231, H402232, H402233, H402234, H402290, H402291, H402292, H402293, H402294, H40231, H40232, H40233, H40239, H40241, H40242, H40243, H40249, H4030X0, H4030X1, H4030X2, H4030X3, H4030X4, H4031X0, H4031X1, H4031X2, H4031X3, H4031X4, H4032X0, H4032X1, H4032X2, H4032X3, H4032X4, H4033X0, H4033X1, H4033X2, H4033X3, H4033X4, H4040X0, H4040X1, H4040X2, H4040X3, H4040X4, H4041X0, H4041X1, H4041X2, H4041X3, H4041X4, H4042X0, H4042X1, H4042X2, H4042X3, H4042X4, H4043X0, H4043X1, H4043X2, H4043X3, H4043X4, H4050X0, H4050X2, H4050X3, H4050X4, H4051X2, H4051X3, H4051X4, H4052X2, H4052X3, H4052X4, H4053X2, H4053X3, H4053X4, H4060X0, H4060X1, H4060X2, H4060X3, H4060X4, H4061X0, H4061X1, H4061X2, H4061X3, H4061X4, H4062X0, H4062X1, H4062X2, H4062X3, H4062X4, H4063X0, H4063X1, H4063X2, H4063X3, H4063X4, H40811, H40812, H40813, H40819, H40821, H40822, H40823, H40829, H40831, H40832, H40833, H40839, H4089, H409, H42, Q150 |

Supplementary Table 2. The detailed case number classified based on the diagnostic code (ICD-9-CM) of the retinal disease before the diagnosis of Parkinson’s disease (PD).

| **ICD-9-CM of the included retinal diseases** | **Non-PD** | **PD** |
| --- | --- | --- |
| 362.5 Degeneration of macula and posterior pole of retina | 1416 | 402 |
| 362.0 Diabetic retinopathy | 877 | 268 |
| 362.3 Retinal vascular occlusion | 304 | 67 |
| 362.1 Other background retinopathy and retinal vascular changes | 214 | 60 |
| 362.6 Peripheral retinal degenerations | 125 | 34 |
| 362.8 Other retinal disorders | 122 | 34 |
| 361.0 Retinal detachments and defects | 82 | 17 |
| 362.9 Unspecified retinal disorder | 65 | 90 |
| 362.4 Separation of retinal layers | 59 |  |
| 361.3 Retinal defects without detachment | 49 |  |
| 361.9 Unspecified retinal detachment | 32 |  |
| 361 Retinal detachments and defects | 83 |  |
| 362 Other retinal disorders |  |  |
| 361.1 Retinoschisis and retinal cysts |  |  |
| 361.2 Serous retinal detachment |  |  |
| 361.8 Other forms of retinal detachment |  |  |
| 362.2 Other proliferative retinopathy |  |  |
| 363.0 Focal chorioretinitis and focal retinochoroiditis |  |  |
| 363.1 Disseminated chorioretinitis and disseminated retinochoroiditis |  |  |
| 363.2 Other and unspecified forms of chorioretinitis and retinochoroiditis |  |  |
| 363.3 Chorioretinal scars |  |  |
| 363.8 Other disorders of choroid |  |  |
| 363.9 Unspecified disorder of choroid |  |  |

Supplementary Table 3. The detailed case number classified based on the diagnostic code (ICD-9-CM) of the optic nerve disease before the diagnosis of Parkinson’s disease (PD).

| **ICD-9-CM of the included optic nerve diseases** | **Non-PD** | **PD** |
| --- | --- | --- |
| 377.1 Optic atrophy | 184 | 58 |
| 377.4 Other disorders of optic nerve | 52 | 14 |
| 377 Disorders of optic nerve and visual pathways | 39 | 14 |
| 377.0 Papilledema |  |  |
| 377.2 Other disorders of optic disc |  |  |
| 377.6 Disorders of other visual pathways |  |  |
| 377.9 Unspecified disorder of optic nerve and visual pathways |  |  |

Supplementary Table 4. The detailed case number classified based on the diagnostic code (ICD-9-CM or ICD-10-CM) of the retinal disease in the follow-up cohort of people with and without Parkinson’s disease (PD).

| **ICD-9-CM and ICD-10-CM of the included retinal diseases** | Non-PD | PD |
| --- | --- | --- |
| 362.5 Degeneration of macula and posterior pole of retina | 919 | 196 |
| 362.0 Diabetic retinopathy | 374 | 69 |
| H353 Degeneration of macula and posterior pole | 391 | 51 |
| 362.3 Retinal vascular occlusion | 136 | 16 |
| E113 Type 2 diabetes mellitus with ophthalmic complications | 117 | 25 |
| 362.1 Other background retinopathy and retinal vascular changes | 90 | 89 |
| 362.8 Other retinal disorders | 77 |  |
| 362.6 Peripheral retinal degenerations | 66 |  |
| 362.9 Peripheral retinal degenerations | 39 |  |
| 361.0 Retinal detachment with retinal defect | 32 |  |
| H350 Background retinopathy and retinal vascular changes | 31 |  |
| 361.3 Retinal defects without detachment | 23 |  |
| H348 Other retinal vascular occlusions | 26 |  |
| H359 Unspecified retinal disorder | 144 |  |
| H358 Other specified retinal disorders |  |  |
| H357 Separation of retinal layers |  |  |
| H356 Retinal hemorrhage |  |  |
| H354 Peripheral retinal degeneration |  |  |
| H342 Other retinal artery occlusions |  |  |
| H340 Transient retinal artery occlusion |  |  |
| H338 Other retinal detachments |  |  |
| H333 Retinal breaks without detachment |  |  |
| H332 Serous retinal detachment |  |  |
| H330 Retinal detachment with retinal break |  |  |
| H32 Chorioretinal disorders in diseases classified elsewhere |  |  |
| H310 Chorioretinal scars |  |  |
| H300 Focal chorioretinal inflammation |  |  |
| G453 Amaurosis fugax |  |  |
| 363.3 Chorioretinal scars |  |  |
| 363.2 Other and unspecified forms of chorioretinitis and retinochoroiditis |  |  |
| 363.0 Focal chorioretinitis and focal retinochoroiditis |  |  |
| 362.4 Separation of retinal layers |  |  |
| 362.2 Other proliferative retinopathy |  |  |
| 361.9 Unspecified retinal detachment |  |  |
| 361.8 Other forms of retinal detachment |  |  |
| 362 Other retinal disorders |  |  |

Supplementary Table 5. The detailed case number classified based on the diagnostic code (ICD-9-CM or ICD-10-CM) of the optic nerve disease in the follow-up cohort of people with and without Parkinson’s disease (PD).

| **ICD-9-CM and ICD-10-CM of the included retinal diseases** | Non-PD | PD |
| --- | --- | --- |
| 377.1 Optic atrophy | 103 | 23 |
| 377.4 Other disorders of optic nerve | 33 | 27 |
| H472 Optic atrophy | 27 |  |
| 377.9 Unspecified disorder of optic nerve and visual pathways | 23 |  |
| H479 Unspecified disorder of visual pathways | 40 |  |
| H476 Disorders of visual cortex |  |  |
| H473 Other disorders of optic disc |  |  |
| H471 Papilledema |  |  |
| H470 Disorders of optic nerve, not elsewhere classified |  |  |
| 377.7 Disorders of visual cortex |  |  |
| 377.6 Disorders of other visual pathways |  |  |
| 377.2 Other disorders of optic disc |  |  |
| 377.0 Papilledema |  |  |
| 377 Disorders of optic nerve and visual pathways |  |  |
